# Mechanical Properties of Cement Mortar Containing Ground Waste Newspaper as Cementitious Material

**DOI:** 10.3390/ma16041374

**Published:** 2023-02-06

**Authors:** Jae-In Lee, Chae-Young Kim, Joo-Ho Yoon, Se-Jin Choi

**Affiliations:** Department of Architectural Engineering, Wonkwang University, 460 Iksan-daero, Iksan 54538, Republic of Korea

**Keywords:** waste newspaper, cement mortar, compressive strength, tensile strength, carbonation depth

## Abstract

In recent years, several studies have reported the recycling of by-products generated by the paper industry and their application to the construction industry. A majority of the existing studies used waste paper sludge ash, and considerable energy is consumed in such incineration processes. This may further contribute to air pollution. In this study, we used waste newspaper (WNP), which underwent a simple crushing process without a separate high-temperature treatment process, and we integrated it in cement mortar. We prepared mortars containing 0%, 0.2%, 0.4%, 0.6%, 0.8%, and 1.0% ground WNP as a cement substitute. Subsequently, the fluidity, compressive strength, tensile strength, carbonation depth, drying shrinkage, and microstructure of the mortars were compared and analyzed. The 28-day compressive strength of the mortar samples with WNP was approximately 3.2–16.1% higher than that of the control sample. The 28-day accelerated carbonation depth of the samples with WNP was approximately 1.03–1.61 mm. Furthermore, their carbonation resistance was approximately 5.2–39.4% higher than that of the control sample. Compressive strength, tensile strength, and carbonation resistance were improved by appropriately using ground WNP as a cement substitute in cement mortar. In this study, the appropriate amount of WNP according to the mechanical properties of cement mortar was found to be 0.4–0.8%, and considering the durability characteristics, the value 0.6 was the most ideal.

## 1. Introduction

In recent years, various efforts have been made to reduce greenhouse gas emissions and environmental load at home and abroad, such as through the use of ecofriendly materials and alternative energy [1,2]. In addition, as countries are promoting carbon-neutral policies, the construction industry must make efforts to reduce greenhouse gas emissions [3]. Portland cement is widely used in the construction industry. It is a powder-type binder that has been used for a long time. It strongly affects environmental pollution, accounting for approximately 5–9% of greenhouse gas emissions generated during cement production [4]. To reduce total cement consumption in the concrete industry, many studies have investigated the application of cementitious materials such as fly ash, blast furnace slag, and silica fume to cement concrete [5,6,7,8,9,10].

Meanwhile, approximately 1.6 million tons of paper sludge, which is a by-product of the paper industry, is generated annually in Korea. This paper sludge contains about 60–70% moisture and causes environmental pollution when it is dumped in landfills, incinerated, and illegally discharged during the treatment process [11].

To solve these problems, several studies have examined the recycling of by-products from the paper industry and their application to the construction industry [12,13,14,15,16,17,18,19,20,21,22]. Sudha et al. [13] reviewed the strength characteristics of concrete mixed with 5%, 10%, 15%, and 20% waste paper sludge ash as a cement substitute. They reported that the compressive strength and tensile strength of concrete increased when it was mixed with up to 10% waste paper sludge ash and decreased thereafter. Shabbir et al. [14] investigated the feasibility of waste paper sludge ash (WPSA) use in concrete. The setting time of cement and the weight of concrete decreased as the mixing ratio of WPSA increased. In addition, WPSA could replace up to 15% of cement by weight. Garcia et al. [15] examined the pozzolanic properties of waste paper sludge. They reported high pozzolanic activity when paper ink-removed sludge was calcined at 700 °C. Additionally, compressive strength increased when 10% of the ink-removed sludge was used as a cement substitute. Hong et al. [16] used paper sludge ash (PSA) to produce powder and then mixed it with concrete. They found that the water absorption and thermal conductivity of the sample with 12% PSA were 84% and 86% of the non-blended sample, respectively. Bui et al. [17] used by-products such as waste paper sludge ash (PSA), fly ash, and silica fume in recycled aggregate concrete. The outcomes of their study reported that PSA improved the mechanical properties of recycled aggregate concrete at an early age.

However, a majority of the existing studies used waste paper sludge ash. In the associated incineration process, a significant amount of energy is consumed, which may further contribute to air pollution. In this study, we prepared waste newspaper (WNP), which underwent a simple crushing process without a separate high-temperature treatment process, and incorporated it into cement mortar. We conducted an experiment to study the applicability of ground WNP as a cement substitute. For this purpose, we prepared mortars containing 0%, 0.2%, 0.4%, 0.6%, 0.8%, and 1.0% ground WNP for use as a cement substitute. We then analyzed and compared the fluidity, compressive strength, tensile strength, carbonation depth, drying shrinkage, and microstructure of the mortars.

## 2. Experimental Materials and Methods

### 2.1. Materials

Ordinary Portland cement (Hanil Hyundai Cement, Seoul, Republic of Korea) was used in this study. Natural sand from Namwon with a density of 2.60 g/cm^3^ and a fineness modulus of 2.45 was used as the fine aggregate. We used WNP available in the Wonkwang University, Iksan-si, Republic of Korea. Based on the outcomes of several preliminary experiments, it was submerged in water for 24 h, dried for 48 h, and then grinded using a grinder.

**Figure 1:**
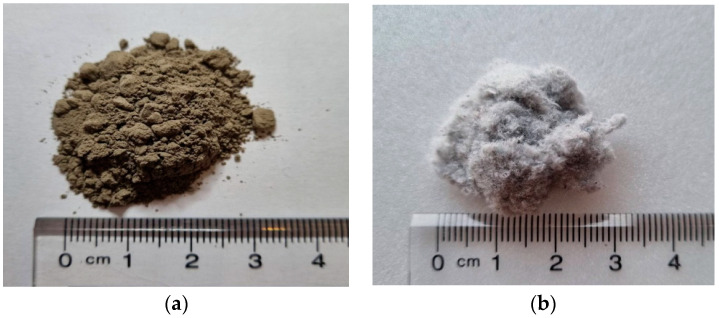
Photographs of (**a**) cement and (**b**) ground waste newspaper (WNP).

**Figure 2:**
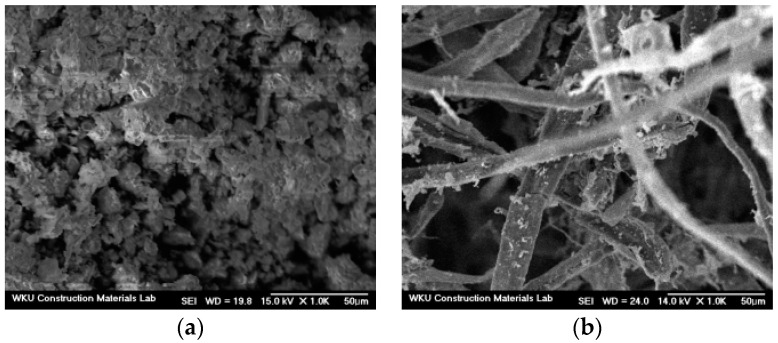
Scanning electron microscopy (SEM) images of (**a**) cement and (**b**) ground WNP.

**Figure 3:**
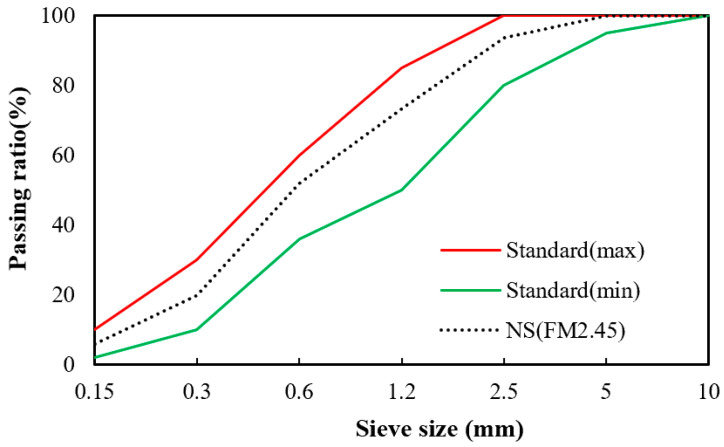
Particle size distribution of fine aggregates (NS: natural sand).

Table 1 and Table 2 show the chemical composition of the cement and WNP, respectively. Table 3 shows the physical properties of the fine aggregate. Figure 1 and Figure 2 show the shapes and scanning electron microscopy (SEM) images of the cement and ground WNP, and Figure 3 shows the particle size distribution curve of the fine aggregate.

### 2.2. Mixing Proportions and Testing Methods

Table 4 shows the mixing proportions of the cement mortars used in this study. We selected W/B 50%, which is widely used as a water–binder ratio (W/B) for normal concrete mixtures in Korea. We used ground WNP to replace 0%, 0.2%, 0.4%, 0.6%, 0.8%, and 1.0% of the cement by weight. Cubic specimens with dimensions of 50 mm × 50 mm × 50 mm were prepared via molding for compressive strength testing, and cylindrical specimens with dimensions of ø50 mm × 100 mm were prepared for split tensile strength testing [23] and carbonation tests. In addition, 40 mm × 40 mm × 160 mm specimens were prepared for evaluation of drying shrinkage. We demolded the specimens after 24 h and cured them in a water tank at 20 °C until they reached the required age.

**Figure 4:**
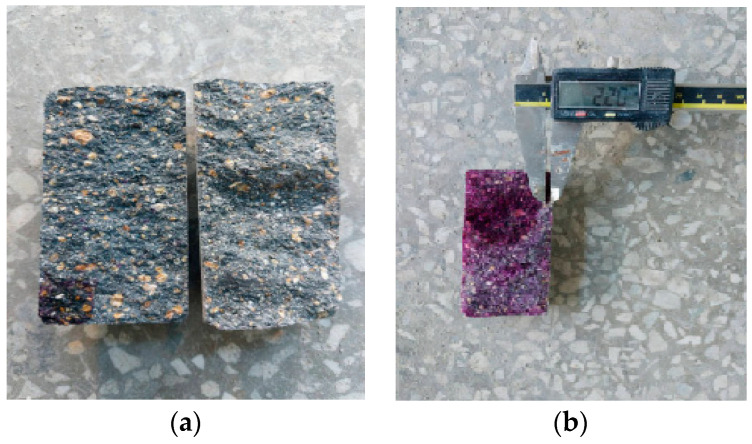
Carbonation depth measurement of (**a**) splitting samples and (**b**) the measurement of carbonation depth.

Mortar flow and compressive strength were measured according to KS L 5105 [24], and tensile strength was determined according to KS F 2423 [25]. In the carbonation test, carbonation depth was measured after the carbonation process using a phenolphthalein solution in an accelerated carbonation chamber according to KS F 2584 [26] (Figure 4). In the case of drying shrinkage, we demolded after 24 h in accordance with KS F 2424 [27] and cured in a water tank at 20 ± 3 °C for 1 week. The samples were then preserved in an environment where the temperature was maintained at 20 ± 3 °C and the humidity at 60 ± 5%, and it was measured using a contact gauge. In addition, SEM (AIS1800C, SERON Technologies, Seoul, Republic of Korea) and energy-dispersive X-ray spectroscopy (EDS; OXFORD Instruments, Xplore, Abingdon, UK) were used for microstructural analysis.

## 3. Results and Discussion

### 3.1. Mortar Flow

**Figure 5:**
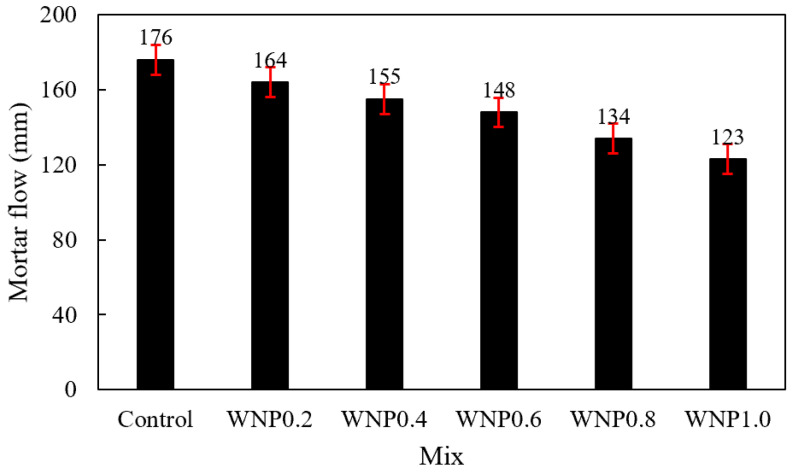
Mortar flow.

Figure 5 shows the change in mortar flow according to the WNP mixing ratio. In the control sample without WNP, the highest flow was approximately 176 mm. In the case of the samples with WNP, the flow was the highest in the WNP0.2 sample (164 mm). Mortar flow gradually decreased as the WNP mixing ratio increased. The flow of the WNP1.0 sample was the lowest at approximately 123 mm, which was approximately 30.1% lower than that of the control sample.

This was because cellulose, which is the main component of WNP, is a hydrophilic material with high water absorption [28,29]. Thus, it absorbed water during mixing and the flow decreased. The decrease in mortar flow may cause deterioration of workability and poor compaction at construction sites.

### 3.2. Compressive Strength

**Figure 6:**
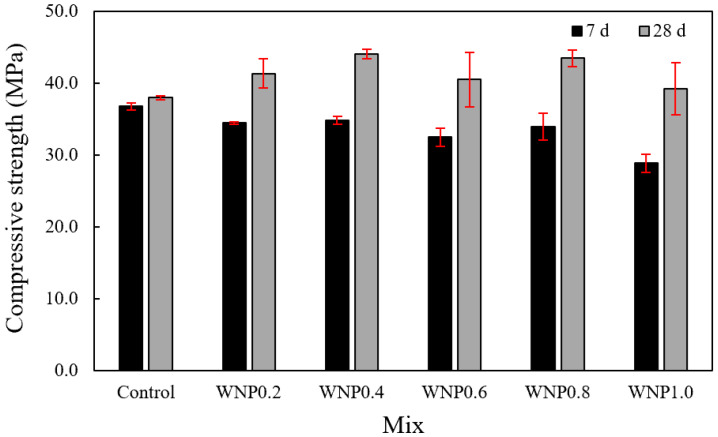
Compressive strength.

Figure 6 shows the change in the compressive strength of the mortars according to the WNP mixing ratio. At an age of 7 days, the highest compressive strength (approximately 36.8 MPa) was observed in the control sample. In the case of the samples with WNP, the highest compressive strength (approximately 34.9 MPa) was observed in the WNP0.4 sample, and the lowest compressive strength (approximately 28.9 MPa) was observed in the WNP 1.0 sample. The 7-day compressive strength of the samples with WNP was approximately 5.1–21.4% lower than that of the control sample.

At an age of 28 days, the compressive strength of the control sample was approximately 38.0 MPa, and that of the WNP0.4 sample was approximately 44.1 MPa, which was approximately 16.1% higher than that of the control sample. The 28-day compressive strength of the WNP0.8 sample was approximately 43.5 MPa, which was approximately 27.9% higher than the 7-day compressive strength. The 28-day compressive strength of all mortar samples with WNP was approximately 3.2–16.1% higher than that of the control sample. In addition, the compressive strength evolution rate of the samples with WNP after 7 days was 19.9–35.9%, which was significantly higher than that of the control sample (3.2%). The compressive strength of the samples with WNP increased because the water–binder ratio decreased as WNP absorbed water during mixing. In addition, the hydration product adhered to the rough cellulose surface and increased the density of the internal structure [30].

In this study, the early compressive strength of the sample that used WNP was lower than that of the control sample, and the strength of the sample increased afterwards. This may be attributed to the fact that initial hydration was delayed due to the high water absorption of WNP and the increase in micropores [16,31]. In addition, the tendency to increase strength may be attributed to the release of absorbed moisture, which thus promotes hydration; hydration products may form on the cellulose surface to fill pores, resulting in a dense structure [30,32]. However, when the amount of WNP used increases, some strength may be lost due to the fiber ball phenomenon of WNP, which is similar to fiber [33].

### 3.3. Split Tensile Strength

**Figure 7:**
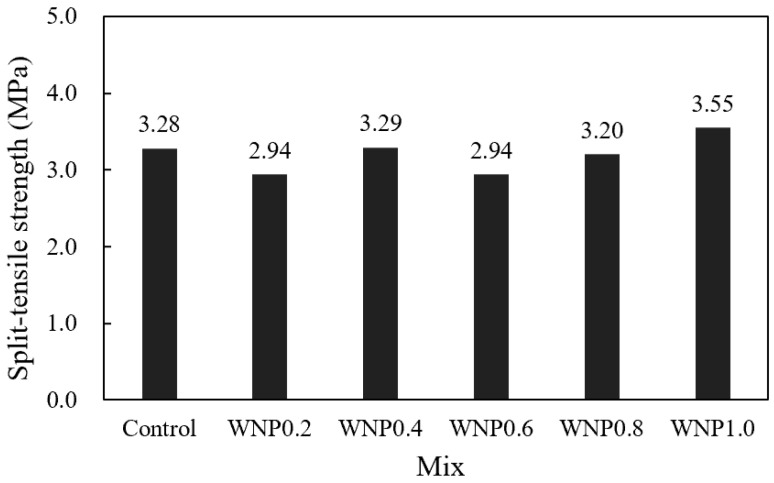
Split tensile strength.

Figure 7 shows the change in the split tensile strength of the mortar according to the WNP mixing ratio after 28 days. The tensile strength of the control sample was approximately 3.28 MPa. The tensile strength of the WNP0.4 sample was approximately 3.29 MPa, which was similar to that of the control sample. The tensile strength of the WNP1.0 sample was approximately 3.55 MPa, which was the highest, and it was approximately 8.2% higher than that of the control sample.

The increase in the tensile strength of mortar samples containing WNP may be attributed to the improvement in adhesion performance between the rough surface of WNP and the cement paste [34,35]. However, in some samples that contain WNP, strength degraded due to the fiber ball phenomenon [33], indicating the need for further research on the correlation between the homogeneous dispersion method and mechanical properties.

### 3.4. Carbonation Resistance

**Figure 8:**
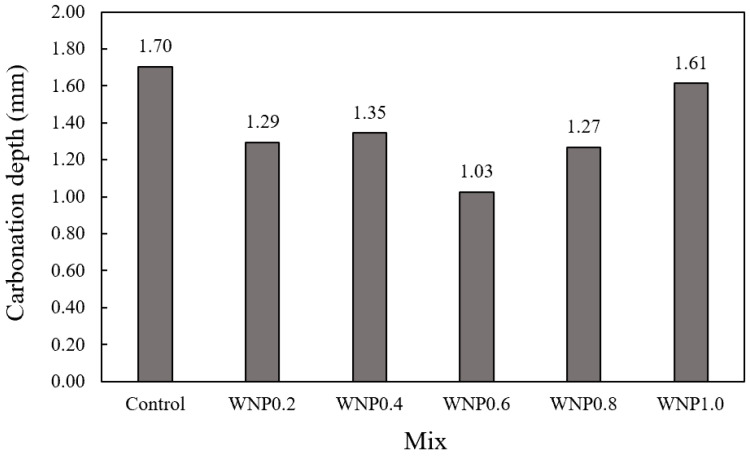
Carbonation resistance.

Figure 8 shows the change in the carbonation depth of the mortar samples according to the WNP mixing ratio. The accelerated carbonation depth of the control sample was the highest at approximately 1.70 mm. The carbonation depth of the WNP0.6 sample was the lowest at approximately 1.03 mm, and it was approximately 39.4% lower than that of the control sample. The carbonation depth of the WNP1.0 sample was approximately 1.61 mm, which was the highest among the samples with WNP. This was due to the relatively low 28-day compressive strength [36] of the WNP1.0 sample, as shown in the compressive strength test results (Figure 5). Carbonation depth did not exhibit a variation trend according to the WNP mixing ratio. In addition, the carbonation depth of all the samples with WNP was lower than that of the control samples. The 28-day accelerated carbonation depth of the samples with WNP was approximately 1.03–1.61 mm, and their carbonation resistance was approximately 5.2–39.4% higher than that of the control sample. This was because cellulose formed an asymmetric structure, which reduced the inflow rate of CO_2_ through internal crack control, thereby increasing carbonation resistance [37].

**Figure 9:**
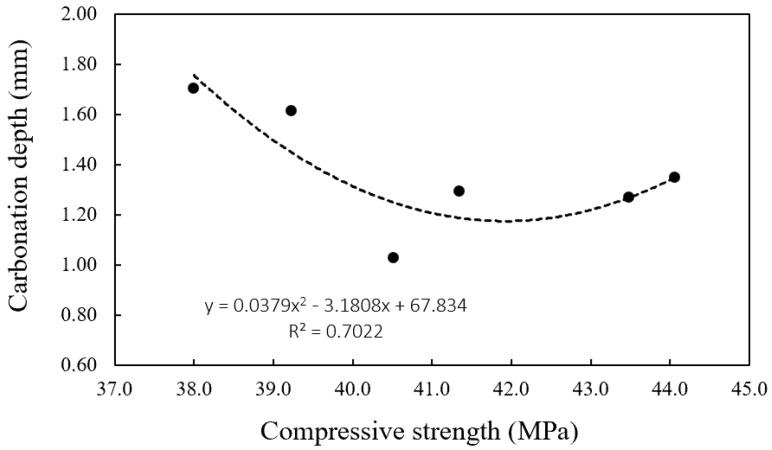
Correlation between compressive strength and carbonation depth.

Figure 9 shows the correlation between compressive strength and carbonation depth. Carbonation depth tends to decrease as compressive strength increases, and the correlation was not sufficiently high.

### 3.5. Drying Shrinkage

**Figure 10:**
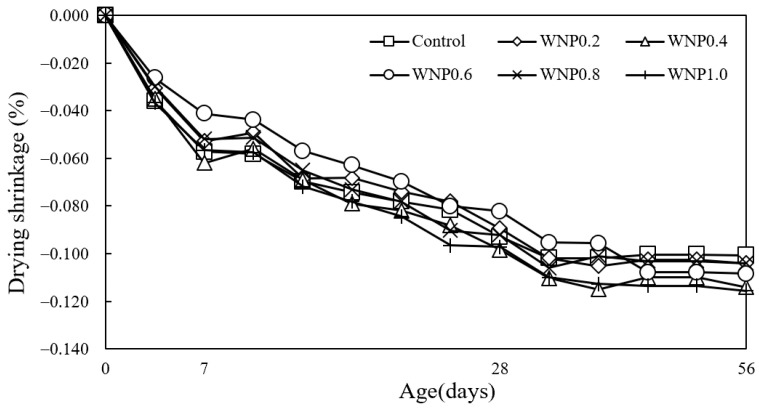
Drying shrinkage.

Figure 10 shows the change in mortar drying shrinkage according to the WNP mixing ratio. The drying shrinkage of the control sample was the lowest at approximately 0.101%. The drying shrinkage of the WNP0.2 and WNP0.8 samples was approximately 0.104%, which was relatively low. The drying shrinkage of the WNP1.0 sample was the highest at approximately 0.116%.

The drying shrinkage of the samples with WNP was higher than that of the control sample because cellulose absorbed moisture and then released the absorbed water over time [38]. According to the existing literature [39,40], approximately 30–40% of cellulose can be extracted from newspaper.

### 3.6. Microstructural Analysis

**Figure 11:**
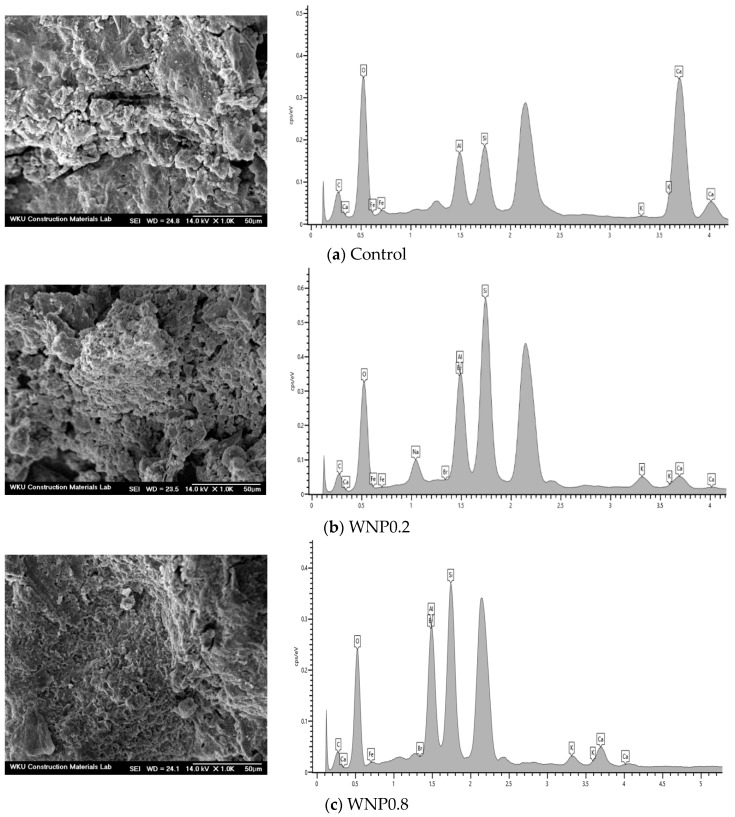
Microstructural analysis (SEM and energy-dispersive X-ray spectroscopy) results for (**a**) control, (**b**) WNP0.2, and (**c**) WNP0.8 samples.

Figure 11 shows the microstructural analysis of the samples. From the SEM images, it can be observed that there was no significant difference between the surface of the sample containing WNP and the surface of the control sample.

Table 5 shows the EDS results for component analysis of the samples. The components were detected in the order of Ca, C, Si, and Al in the control sample and C, Si, Al, and Ca in the WNP0.2 and WNP0.8 samples. The amounts of Si and Al in the samples with WNP were larger than those in the control sample.

## 4. Conclusions

The main results of the study are summarized below.

1. Mortar flow gradually decreased as the WNP mixing ratio increased. This may be attributed to the fact that cellulose, which is the main component of WNP, is a hydrophilic material with high water absorbency. Thus, it absorbed water during mixing and the flow subsequently decreased.
2. The early compressive strength of the sample containing WNP was lower than that of the control sample, and strength increased afterwards. This may be attributed to the fact that initial hydration was delayed due to the high water absorption of WNP and the increase in micropores. In addition, the tendency to increase strength may be caused by the release of absorbed moisture, which thus promotes hydration; hydration products may form on the cellulose surface to fill pores, resulting in a dense structure.
3. The 28-day accelerated carbonation depth of the samples with WNP was approximately 1.03–1.61 mm, and their carbonation resistance was approximately 5.2–39.4% higher than that of the control sample.
4. It was confirmed that compressive strength, tensile strength, and carbonation resistance were improved by utilizing ground WNP as a cement substitute in mortar. In this study, the appropriate amount of WNP according to the mechanical properties of cement mortar was found to be 0.4–0.8%, and considering durability characteristics, 0.6% WNP was the optimal value.

In the future, it is necessary to examine the correlation between the microstructure, mechanical properties, and durability properties of samples with WNP.

## Figures and Tables

**Table 1:** Chemical composition of cement.

Type	SiO_2_	Al_2_O_3_	Fe_2_O_3_	CaO	MgO	K_2_O	Blaine (cm^2^/g)	Density (g/cm^3^)
Ordinary Portland cement	17.43	6.50	3.57	64.40	2.55	1.17	3430	3.15

**Table 2:** Chemical composition of WNP.

Type	C	O	Si	Al	Ca	K
Ground waste newspaper (WNP)	40.6	38.3	9.5	9.0	2.3	0.3

**Table 3:** Physical properties of fine aggregate.

Type	Fineness Modulus	Density (g/cm^3^)	Water Absorption (%)
Natural sand (NS)	2.45	2.60	1.0

**Table 4:** Mixing proportions of mortars.

Mix	W/B (%)	S/a * (%)	WNP ** (C *%)	Water (kg/m^3^)	Cement (kg/m^3^)	NS (kg/m^3^)	WNP (kg/m^3^)
Control	50	42	0	170	340.0	739	0
WNP0.2			0.2		339.3		0.7
WNP0.4			0.4		338.6		1.4
WNP0.6			0.6		338.0		2.0
WNP0.8			0.8		337.3		2.7
WNP1.0			1.0		336.6		3.4

* S/a: sand to aggregate ratio, ** WNP: waste newspaper.

**Table 5:** Energy-dispersive X-ray spectroscopy analysis result.

Mix	Element	Weight (%)	б
Control	O	44.88	55.37
	Ca	24.55	12.09
	C	14.49	23.82
	Si	4.24	2.98
	Al	4.01	2.93
	K	0.26	0.13
WNP0.2	O	38.12	40.52
	Ca	2.82	1.19
	C	28.03	39.68
	Si	16.45	9.96
	Al	8.81	5.55
	K	2.19	0.95
WNP0.8	O	39.5	45.05
	Ca	4.2	1.92
	C	21.4	32.54
	Si	16.0	10.41
	Al	10.7	7.25
	K	1.9	0.86

## Data Availability

Not applicable.
